# Early Reflections on Mphatlalatsane, a Maternal and Neonatal Quality Improvement Initiative Implemented During COVID-19 in South Africa

**DOI:** 10.9745/GHSP-D-22-00022

**Published:** 2022-10-31

**Authors:** Willem Odendaal, Ameena Goga, Terusha Chetty, Helen Schneider, Yogan Pillay, Carol Marshall, Ute Feucht, Tsakane Hlongwane, Shuaib Kauchali, Manala Makua

**Affiliations:** a HIV and Other Infectious Diseases Research Unit, South African Medical Research Council, Cape Town, South Africa.; b Department of Psychiatry, Stellenbosch University, Cape Town, South Africa.; c Department of Paediatrics and Child Health, University of Pretoria, Pretoria, South Africa.; d School of Public Health and the South African Medical Research Council Health Services to Systems Research Unit, University of the Western Cape, Cape Town, South Africa.; e Clinton Health Access Initiative, Pretoria, South Africa.; f Department of Global Health, Stellenbosch University, Stellenbosch, South Africa.; g National Department of Health, Pretoria, South Africa.; h Research Centre for Maternal, Fetal, Newborn and Child Health Care Strategies, University of Pretoria, Pretoria, South Africa.; i Maternal and Infant Health Care Strategies Research Unit, South African Medical Research Council, Pretoria, South Africa.; j Department of Obstetrics and Gynaecology, University of Pretoria, Pretoria, South Africa.; k Maternal, Adolescent and Child Health Institute, Durban, South Africa.

## Abstract

Despite global progress in reducing maternal and neonatal mortality and stillbirths, much work remains to be done to achieve the Sustainable Development Goals. Reports indicate that coronavirus disease (COVID-19) disrupts the provision and uptake of routine maternal and neonatal health care (MNH) services and negatively impacts cumulative pre-COVID-19 achievements. We describe a multipartnered MNH quality improvement (QI) initiative called Mphatlalatsane, which was implemented in South Africa before and during the COVID-19 pandemic. The initiative aimed to reduce the maternal mortality ratio, neonatal mortality rate, and stillbirth rate by 50% between 2018 and 2022. The multifaceted design comprises QI and other intervention activities across micro-, meso-, and macrolevels, and its area-based approach facilitates patients’ access to MNH services. The initiative commenced 6 months pre-COVID-19, with subsequent adaptation necessitated by COVID-19. The initial focus on a plan-do-study-act QI model shifted toward meeting the immediate needs of health care workers (HCWs), the health system, and health care managers arising from COVID-19. Examples include providing emotional support to staff and streamlining supply chain management for infection control and personal protection materials. As these needs were addressed, Mphatlalatsane gradually refocused HCWs’ and managers’ attention to recognize the disruptions caused by COVID-19 to routine MNH services. This gradual reprioritization included the development of a risk matrix to help staff and managers identify specific risks to service provision and uptake and develop mitigating measures. Through this approach, Mphatlalatsane led to an optimization case using existing resources rather than requesting new resources to build an investment case, with a responsive design and implementation approach as the cornerstone of the initiative. Further, Mphatlalatsane demonstrates that agile and context-specific responses to crises such as the COVID-19 pandemic can mitigate such threats and maintain interventions to improve MNH services.

## Abstract (precis)

A quality improvement initiative for maternal and neonatal health care demonstrates that a responsive intervention design and implementation approach mitigates threats to clinical services during COVID-19.

## Teaser

**Key Findings:**

- Responding to health workers’ and managers’ immediate needs related to coronavirus disease (COVID-19), followed by reprioritization and inclusion of a newly developed COVID-19 risk matrix, helped Mphatlalatsane staff to identify and mitigate disruptions to public sector maternal and neonatal health care (MNH) services in South Africa.
- Mphatlalatsane demonstrates that agile and context-specific responses to crises, such as the COVID-19 pandemic, can mitigate such threats and maintain interventions to improve MNH services.

**Key Implications:**

- Multifaceted MNH intervention programs are challenging to design and implement, but if they are locally responsive they can benefit health care workers, managers, and patients in a systematic way.
- Program managers must remain alert to the continued disruptions by crises such as COVID-19 to routine MNH care services. A responsive approach requires attention to the immediate needs of health workers, such as emotional support, while balancing this with the ability of staff to identify and mitigatedisruptions to routine services caused by health crises.

## BACKGROUND

### Maternal and Neonatal Mortality and Stillbirths: Globally and in South Africa

Globally, progress has been made over the past 2 decades to reduce maternal mortality, defined as death of a woman during pregnancy or until 42 days post-delivery.^1^ Between 2000 and 2017, the maternal mortality ratio (MMR; number of maternal deaths per 100,000 live births) declined by an estimated 38% (from 342/100,000 to 211/100,000).^2^ Parallel to this improvement, progress is being made in reducing the neonatal mortality rate (NMR; number of live-born infants dying in the first 28 days of life per 1000 live births).^3^ Globally, the NMR decreased from 31 in 2000 to 18 in 2018.^4^ The global stillbirth rate (number of babies born with no signs of life per 1,000 live births)^3^ decreased from approximately 21 in 2000 to about 14 in 2020.^5^ Although evidence of the clinical effects of the coronavirus disease (COVID-19) on mothers and babies is evolving,^6^^,^^7^ a decline in uptake and provision of maternal and neonatal health (MNH) services has been reported globally. This decline includes a 60% and 100% drop, respectively, in antenatal care and family planning facility visits in Bangladesh^8^ and a 50% decrease in the weekly number of institutional births in Nepal.^9^ In Italy, 23% of maternal and perinatal facilities were understaffed during the first 3 months of the COVID-19 outbreak.^10^

Similar to international trends, the pre-COVID-19 MMR in South Africa (SA) decreased from 200/100,000 in 2012 to 134/100,000 in 2017,^11^ albeit with provincial differences. Despite a substantial decline in the MMR, much work still needs to be done to achieve the Sustainable Development Goal for an MMR of less than 70/100,000 by 2030.^12^ The NMR in SA remained stagnant at 12/1,000 live births between 2012 and 2017,^11^ well above the SA National Department of Health (NDOH) 2030 goal of 7/1,000.^13^ No recent data were found on the stillbirth ratios, estimated at 20.2 per 1,000 live births in 2016.^14^ Early indications of the COVID-19 impact in SA show severe adverse effects, both in the epidemiology of MMR, NMR, and stillbirths and in the uptake of maternal services. In comparing the April 2020–March 2021 COVID-19 period with the same 2019–2020 pre-COVID-19 period, Pattinson et al. reported a 40% increase in maternal deaths, with a 3% and 10% increase in neonatal mortality and stillbirths, respectively.^15^ Antenatal care services were generally maintained, but the uptake of reproductive health services decreased by 17% and contraceptive prescriptions by 5%. These numbers are confirmed by Pillay et al., who also found a 3.7% increase in deliveries in public health facilities from March to December 2020.^16^

Early indications of the COVID-19 impact in SA show severe adverse effects, both in MMR, NMR, and stillbirths and in maternal services uptake.

### Improving Maternal, Neonatal, and Child Health Services

Global evidence suggests that microlevel interventions (defined as interventions at the health facility, health care worker, and patient levels) can decrease the MMR, NMR, and stillbirth ratios. Successful interventions include birth preparedness and complication readiness programs^17^; community-based campaigns to strengthen preventative and caregiving practices such as family planning, facility-based births, and immunization uptake^18^^–^^21^; improving women’s educational level^22^; and quality improvement (QI) initiatives.^23^^–^^25^ QI programs capacitate health care workers (HCWs) to unlock service delivery bottlenecks under their control,^26^^,^^27^ rather than focusing on large-scale, resource-intensive changes.^28^^–^^30^ However, as noted by the Lancet Global Health Commission^31^ and others,^32^^,^^33^ microlevel successes can only be sustained and successfully taken to scale if embedded in well-functioning, supportive health systems beyond the microlevel.

In SA, examples of successful microlevel MNH interventions include the Essential Steps in Managing Obstetric Emergencies, a skills-and-drills program for maternity staff, which reduced institutional MMR by 29%^34^; the national prevention of mother-to-child HIV transmission program which reduced early (4–8 weeks postpartum) vertical HIV transmission at 10 weeks to 0.7% in 2018/2019^35^; and the World Health Organization’s 10 steps for the management of severe acute malnutrition, which reduced in-patient mortality among severely malnourished children by up to 39%.^36^

Measured against Sustainable Development Goal targets, the SA rate of adverse birth outcomes, such as stillbirths and preterm births,^37^ is still unacceptably high, given international consensus that these are preventable through improved quality of care.^38^^–^^41^ Experts postulate that approximately 60% of maternal deaths and up to 50% of neonatal deaths are potentially preventable.^34^^,^^42^ This corresponds with data modeling from 81 low- and middle-income countries, showing that approximately 25% of neonatal deaths and stillbirths between 2016 and 2020 were preventable through improved quality of pre- and postnatal care provided to women and neonates.^43^

In this field action report, we describe and reflect on a QI initiative called Mphatlalatsane (meaning “the bright star before dawn”), which aimed to strengthen the MNH service package across all levels of care. We detail the goal, implementation context, key design features, and adaptations during the first 19 months of the COVID-19 pandemic, from March 2020 to October 2021.

Mphatlalatsane aimed to strengthen the MNH service package across all levels of care to reduce maternal and neonatal mortality and stillbirths in South Africa.

## MPHATLALATSANE

### Goal and Implementation Context

**FIGURE 1:**
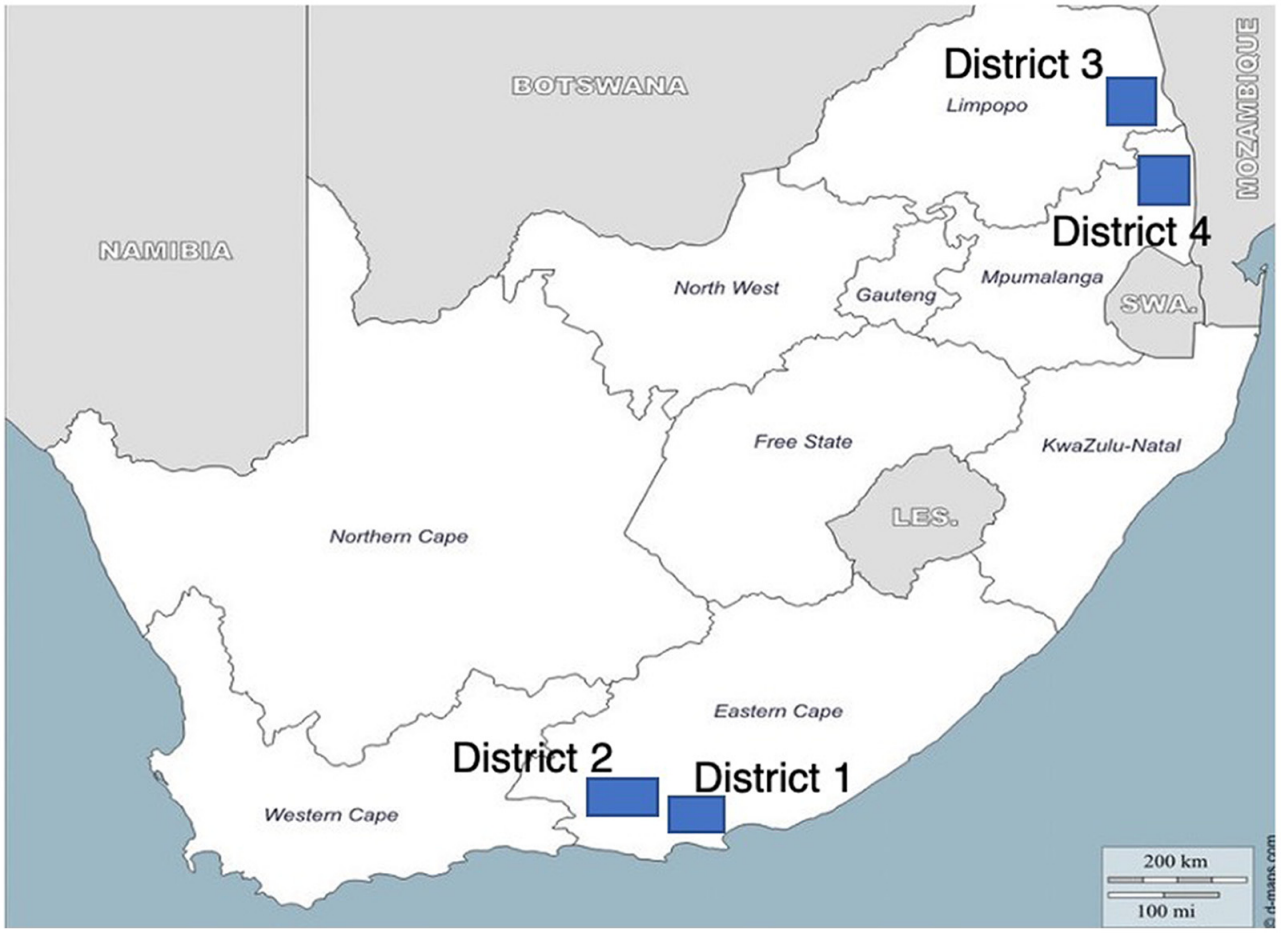
Map of Implementation Districts for the Mphatlalatsane Maternal and Newborn Health Quality Improvement Initiative, South Africa

Mphatlalatsane is implemented by the NDOH in 4 purposively selected health districts across the Eastern Cape, Limpopo, and Mpumalanga provinces (Figure 1). The goal is to reduce institutional maternal and neonatal mortality and stillbirths by up to 50% from 2018 to 2022 in 7 selected facilities in each province. The districts and facilities were purposively selected as sites that (1) exhibit below-average MNH service performance and capacity, (2) reflect clinical referral pathways, and (3) represent urban and rural settings. District 1 has a 91% urban population^44^; in District 2, only 27% of the population is urbanized^44^; and in Districts 3 and 4, 81%^45^ and 70%^46^ of the respective populations live in rural areas. In Table 1, we compare the 4 districts against other socioeconomic and health indicators.^47^^–^^53^

**TABLE 1.** Socioeconomic and Health Indicators of the South African Health Districts Served by Mphatlalatsane

**Province**	**Eastern Cape**		**Limpopo**	**Mpumalanga**
	**District 1**	**District 2**	**District 3**	**District 4**
Population estimates,^a^ No.	1,263,051^47^	527,062^48^	138,760^45^	1,743,182^49^
Unemployment rate,^a^ %	29^50^	30^48^	39^45^	37^51^
Health facilities				
Tertiary	2	0	0	1
Regional	1	0	1	2
District	1	10	6	8
CHC	9	3	8	15
PHC clinic	39^52^	59^52^	97^53^	108^49^
Institutional maternal mortality ratio (per 100,000 live births),^b^ 2018^c^		107^13^	106^13^	111^13^
Live births in facility,^a^ No.	18,808^52^	5,909^52^	31,268^45^	40,734
Neonatal mortality ratio (per 1,000 live births)^b^		12.3^52^	12.2^13^	10.8^13^

Abbreviations: CHC, community health center; PHC, primary health care.

^a^ 2018/2019.

^b^ 2018.

^c^ Per province.

### Antecedents and Set-Up Phase

Aligned with the SA Maternal, Perinatal and Neonatal Health Policy,^54^ Mphatlalatsane builds upon a decade of national programs to strengthen maternal, neonatal, and child health services in the country.

- Recommendations from NDOH ministerial committees such as the National Confidential Enquiry into Maternal Deaths Committee since 1997^42^ and the National Perinatal Mortality and Morbidity Committee established in 2008^14^
- The national primary health care reengineering strategy,^55^^,^^56^ with its district clinical specialist teams, to strengthen the clinical governance of maternal, neonatal, and child health,^56^ commencing around 2010
- Standardised Essential Steps in Managing Obstetric Emergencies drills,^57^ commencing in 2008
- The Perinatal Problem Identification Programme and Child Healthcare Problem Identification Programme surveillance and response systems implemented since the 1990s^58^
- Several health system strengthening workshops with managers and clinicians in the Mphatlalatsane districts over the past 10 years

In July 2018, at the commencement of Mphatlalatsane, the management team conducted site visits, engaged with senior managers in the 3 provincial governments (macrolevel), and held planning workshops with the district and subdistrict staff (mesolevel) and facility staff (microlevel). These consultations resulted in a proposal, codesigned with staff, that details implementation activities and partner roles and responsibilities (Table 2). All Mphatlalatsane interventions (example in Box) fall within the NDOH’s authority and are implemented by national, provincial, or district health departments as per their mandate.

**TABLE 2.** Mphatlalatsane Partnerships

**Partner**	**Role and Responsibilities**
South Africa National Department of Health	Custodian: provides strategic focus
Clinton Access Health Initiative	Implementation secretariat: coordinate and direct day-to-day activities; provides technical support through quality improvement advisors
University of Pretoria/South African Medical Research Council Research Centre for Maternal, Fetal, Newborn and Child Health Care Strategies	Content expert: provides input and support for maternal health care strengthening activities
University of Limpopo Trust Initiative for Newborn Care	Content expert: provides input and support for neonatal health care strengthening activities
Institute for Healthcare Improvement	Provides technical support to advisors and quality improvement training (short-term)
South African Medical Research Council and University of the Western Cape	Evaluation team

Consultations resulted in a proposal, codesigned with staff, that details implementation activities and partner roles and responsibilities.

### Ethical Approval

The NDOH commissioned the South African Medical Research Council and University of the Western Cape to evaluate Mphatlalatsane. They received ethical approval (EC019-11/2019) from the South African Medical Research Council Ethics Committee.

### Implementation

Implementation commenced in September 2019 when facility HCWs were trained as QI team leaders. Upon return, these leaders established a QI team in their respective facilities.

Mphatlalatsane management appointed 3 QI advisors, 1 per province, to provide technical support and mentoring to the QI teams. To ensure sustainability, the advisors are required to identify and mentor a provincial official in each province over time. This will embed the QI system within the existing structures of the 3 provincial health departments. The first 2 COVID-19 waves and lockdowns severely disrupted QI team functioning and MNH services, with MNH staff either moved to other posts or too busy dealing with COVID-19–related functions. Staff attrition was a reality in most facilities, in some cases due to COVID-19 fatalities and illness, which required training of new team members. Additional district management support to engage with Mphatlalatsane activities was also needed. With the lifting of travel restrictions since July 2020, the advisors resumed in-person support visits. They were warmly welcomed back, and the teams readily reengaged with their QI activities, over time regaining much of their pre-COVID-19 momentum.

### Key Design Features and Illustrative Examples

#### A Multifaceted Initiative

Mphatlalatsane is multifaceted in several ways. First, it is a multipartnered initiative that seeks synergy between governmental and nongovernmental agencies to improve MNH services. A Program Management Committee (PMC) was established among the 5 partners to meet monthly and serve as a governance and implementation coordination platform.

**FIGURE 2:**
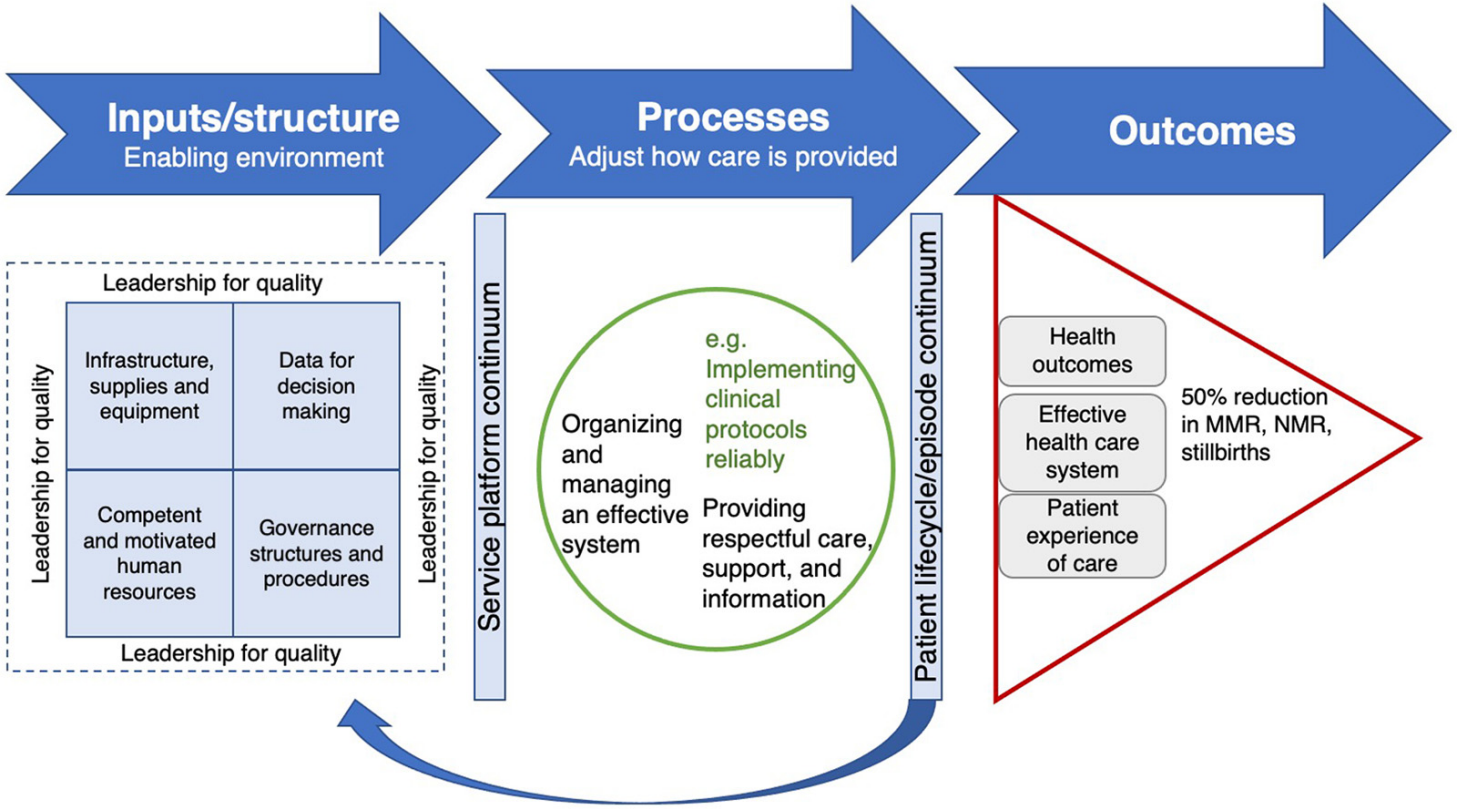
Mphatlalatsane Theory of Change Abbreviations: MMR, maternal mortality ratio; NMR, newborn mortality ratio.

Second, it is multifaceted in its holistic health system strengthening focus. It recognizes that its microlevel intervention of QI for HCWs can only succeed if it is treated as part of the health system ecology: what happens at one level shapes and is shaped by what happens at the other levels. This holistic approach and acknowledgment of the interdependency are evident in its theory of change (Figure 2). The PMC envisages that program outputs and outcomes (triangle) will result from improved service delivery processes (circle) through more efficient use of existing human, infrastructural, and supply chain resources (square).

Third, this integrated approach translates into a portfolio of QI and other types of interventions. At the microlevel, the Mphatlalatsane philosophy advocates agency for HCWs through QI methodologies that upskill them to solve problems within their sphere of influence (Box). Several other activities are underway at meso- and macrolevels to improve governance and leadership. One such activity is enhancing systems of real-time mortality surveillance and associated governance structures to ensure coordinated responses, referred to as monitoring and response units at the subdistrict and district levels.^59^^,^^60^

BOX **Change Idea Developed and Adopted in a Mphatlalatsane Primary Health Care Clinic**

**Problem**

- The clinic’s routine data showed only 63% of first antenatal care (ANC) visits occurred before 20 weeks of gestation, leading to late identification and management of pregnancy complications.

**Aim**

- To improve first ANC visits before 20 weeks’ gestation to 70% within 2 months.

**Implementing a basket of change ideas**

- Irrespective of the reason they visited the clinic, all women of childbearing age were tested for pregnancy; this does not require formal written consent from the patient as verbal explanation of the procedure is the standard of care.
- Community health care workers added pregnancy screening to their services.
- During routine health talks at the clinic, women were encouraged to visit the clinic as soon as a pregnancy was suspected.
- Family planning services were offered to schoolgirls. Girls could either visit the clinic before school, offered as a fast lane, or in the afternoons as part of the clinic’s adolescent and youth friendly services.

**Effectiveness of change idea**

- After 5 weeks of implementation, there were 12 newly diagnosed pregnancies of which 11 were earlier than 20 weeks’ gestation, resulting in 92% of first ANC visits done before 20 weeks’ gestation.
- The first ANC visits before 20 weeks’ gestation improved from 61% to 83% between February 24, 2020 and April 30, 2020.

Mphatlalatsane advocates agency for HCWs through QI methodologies that upskill them to solve problems within their sphere of influence.

Synergistic strengthening of all health system components is central to the intervention portfolio. As an example, the HCWs in Facility A identified improved ward infection control (microlevel) as their QI project. Since they lacked the supplies, the Clinton Health Access Initiative assisted them by streamlining the district-level (mesolevel) supply chain management of hygiene materials. Other examples include engaging with district and provincial (macrolevel) information management staff to strengthen the quality and use of routine data (facilitated by the University of Pretoria/South African Medical Research Council Research Centre for Maternal, Fetal, Newborn and Child Health Care Strategies); the developing and training staff on an instrument to monitor and evaluate the prevalence and management of hypertensive disorders in pregnancy, as well as ensuring that the necessary medical supplies are in stock; establishing an obstetrics response team to drive maternal and perinatal improvement in 1 of the provinces (coordinated by the University of Limpopo Trust Initiative for Newborn Care); and providing technical and logistical support from all partners to the NDOH in drafting a national integrated maternal, perinatal, and neonatal health policy.

#### An Area-Based Initiative

**FIGURE 3:**
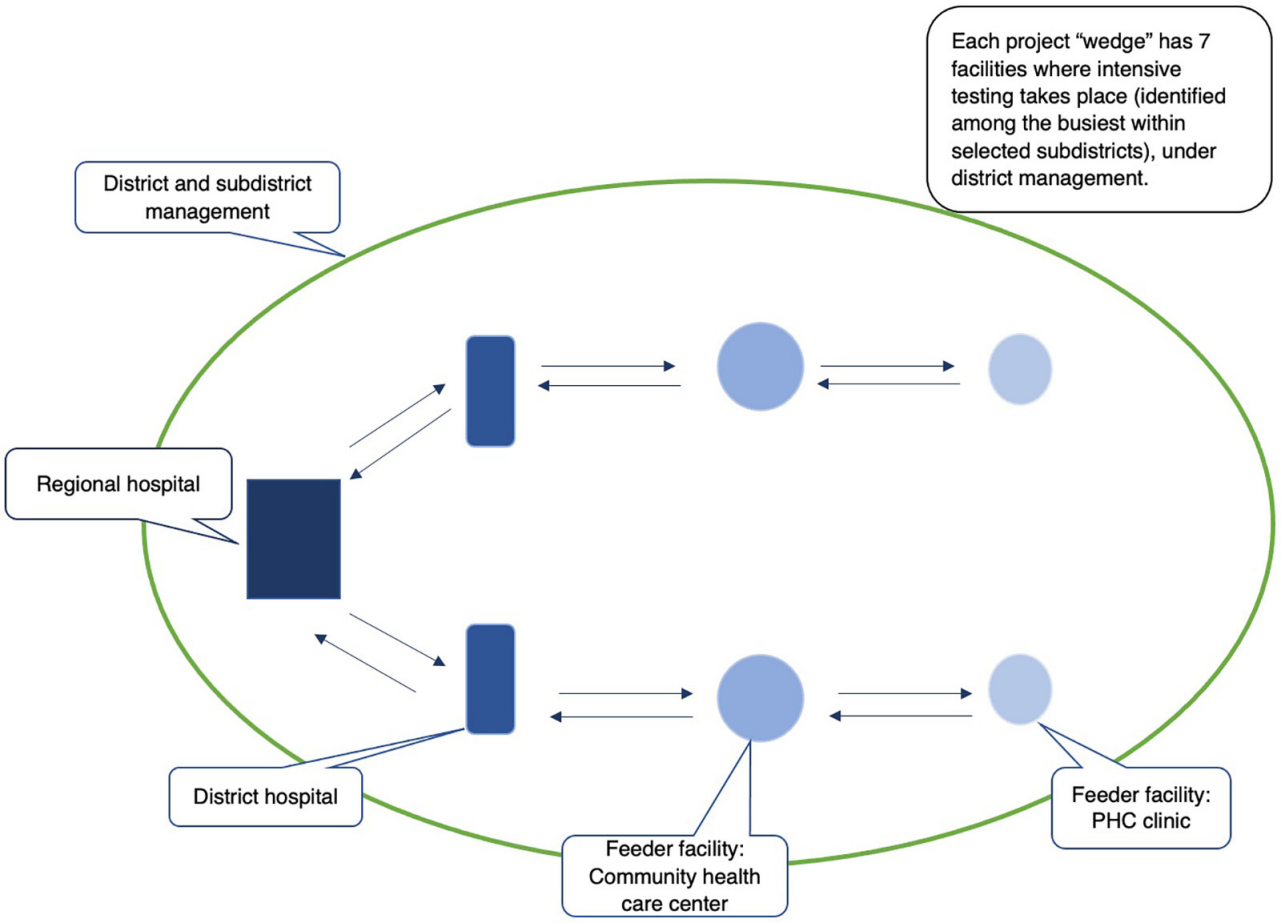
Mphatlalatsane Implementation Wedge in Eastern Cape, Limpopo, and Mpumalanga Provinces, South Africa Abbreviation: PHC, primary health care.

The NDOH identified an “implementation wedge” (Figure 3), comprising 7 selected facilities in each province. The wedge facilities form an integrated referral health system that reflects how patients access services. The wedge includes 2 primary health care clinics feeding into community health centers, which then feed into district hospitals and a regional hospital.

#### A Responsive Approach

Mphatlalatsane’s planning and implementation centers on innovation and responsiveness to local contexts and changing priorities. For example, the PMC adapted the classical QI methodology to an agile model that allows HCWs to choose how their team functions—short-circuiting plan-do-study-act cycles where there is a need to respond to other immediate challenges and allowing teams to have fewer, and more prolonged, plan-do-study-act cycles compared to the standard multiple, rapid cycles. The latter arrangement addressed the reality that the COVID-19 pandemic caused some QI team members to be redeployed to meet non-MNH COVID-19 emergencies. The PMC also implemented other measures in response to the pandemic (Table 3). At the start of the pandemic, they decided to not push ahead with the QI work, but prioritized meeting management structures’ and HCWs’ immediate needs arising from the pandemic. This included providing real-time COVID-19 information through phone calls, WhatsApp groups, and online webinars made available on an existing NDOH digital knowledge hub. Pre-COVID-19, this hub was a resource tool for HCWs to access a wide range of information, including national guidelines for MNH services. The webinars the PMC made available on the hub included maternal- and child-specific interventions in the context of COVID-19. The PMC also provided psychological support to HCWs following the loss of colleagues and family by making the contact details of such services available and was instrumental in getting infection prevention and control guidelines for MNH services adapted during the COVID-19 pandemic.

**TABLE 3.** Mphatlalatsane Responsive Measures to Meet COVID-19 Challenges in South Africa

**COVID-19 Challenges**	**Responsive Measures**
MNH staff: distressed and overworked; redeployed	Shifted focus from QI work to management / HCWs’ immediate needs and offered pandemic response guidance Offered psychological support to HCWs Streamlined procurement of personal protective equipment Made new service delivery algorithms and frameworks available through an existing NDOH digital knowledge hub that addressed COVID-19 challenges
Delivery and uptake of routine MNH services disrupted	Risk matrix 1: offered practical recommendations to higher-level management to mitigate service disruption, e.g., used COVID-19 contact tracing to inform communities that MNH services are available Risk matrix 2: supported facility managers to develop risk mitigation measures within the facility, e.g., reconfigured workspaces to minimize contamination
Essential MNH equipment shortages	Facilitated the procurement and distribution of donated equipment through acceptable mechanisms and coordinated staff training to use it

Abbreviations: COVID-19, coronavirus disease; HCW, health care workers; MNH, maternal and neonatal health; NDOH, South Africa National Department of Health; QI, quality improvement.

The PMC realized that dealing with COVID-19 at times came at the expense of MNH patients. This resulted in the development of 2 risk matrices. The first matrix (Supplement Table S1) offered practical suggestions to MNH managers at subdistrict and higher levels to sustain the delivery and uptake of routine MNH services, including ensuring effective communication with HCWs; using COVID-19 contact tracing activities to deliver the message that MNH services are essential services and thus always available; redefining the roles of HCW cadres, such as using lower-cadre nurses to monitor infection prevention and control compliance in the health facility; and maximizing the use of doulas (non-medical persons trained as birth companions) because family members were not allowed into facilities. The second matrix (Supplement Table S2) was developed to alert HCWs of COVID-19–imposed risks along the continuum of MNH services. The QI teams and QI advisors supported facility managers to develop context-specific solutions to deal with these risks, such as reconfiguring work spaces to minimize sharing of equipment. An online, self-paced, 14-module sexual and reproductive health training curriculum was developed to keep HCWs updated on new MNH guidelines. Finally, through the Clinton Health Access Initiative, Mphatlalatsane facilitated the supply of needed equipment through approved donations that abided by the Public Finance Management Act in SA.

The PMC realized that dealing with COVID-19 at times came at the expense of MNH patients.

## REFLECTIONS

Our preliminary reflections concern the 2 design characteristics considered key to the successful implementation of Mphatlalatsane, lessons learned during COVID-19, and thoughts on sustaining the initiative’s successes.

### Key Design Characteristics

#### Inclusivity

Mphatlalatsane focuses on providing support to all cadres of HCWs, including community health workers and managers, across all levels, to improve MNH services. The PMC takes an ecological view,^61^ advocated as systems thinking, and acknowledges that the health system is an open system, comprising many actors and ever-changing processes and contexts that interact with each other and are defined by their interactions.^61^^–^^63^

#### Embeddedness

Mphatlalatsane is designed, implemented, and evaluated as implementation research. This confirms the importance of an ecological view of the systems in which Mphatlalatsane is implemented, as it focuses on exploring what works, for whom, and under which circumstances.^64^ It is embedded in real-world settings and with regular, real-time participant feedback it promotes the integration of new effective practices into standard care.^65^^,^^66^

Embedded in real-world settings, Mphatlalatsane promotes the integration of new effective practices into standard care.

### Implementing MNH Interventions in the Context of Crises Such as COVID-19

The COVID-19 pandemic and its aftereffects on people’s health and the uptake and delivery of health care services will remain a reality for years to come. During similar crises in the future, the health system will need initiatives such as Mphatlalatsane to address the needs of health systems, HCWs, and managers while sustaining and improving MNH. Our implementation experience proves that an agile and responsive design approach makes this possible. Agile and responsive interventions require leadership attuned to HCW vulnerabilities at the point of service delivery, conscious of meso- and macrolevel management needs, with the ability to adapt health system arrangements to maintain its core function of quality care to mothers and neonates.^67^^,^^68^

### Sustainability

Stable and robust leadership must balance the often conflicting demands of people and processes that constitute the health service ecology. Initiatives such as Mphatlalatsane must avoid turning into a collection of fragmented projects and instead forge true synergy between interventions and partners to ensure collective impact and sustainability. While recognizing the need for simultaneous action at multiple levels, our emerging experience is that absorptive capacity for sustainability is highly variable, especially in the context of chronic stresses and multiple, often unrelated, donor initiatives. Finally, public health services are hampered by cultures of compliance and risk aversion, undermining agency among managers and HCWs who then act as gatekeepers rather than gateways for change.

Mphatlalatsane operates under the philosophy that quality care does not begin and end with more resources. It recognizes that known drivers of QI, such as shared decision making, accountability at all levels, and interpersonal relationships^36^^,^^69^^–^^71^ need to remain implementation mantras. The reality that many interventions end when funding ceases requires deliberate efforts to identify and institutionalize good practices beyond Mphatlalatsane facilities. Through this approach, Mphatlalatsane adopted an optimization case using existing resources rather than introducing new approaches to build an investment case.

## CONCLUSION

The effectiveness of Mphatlalatsane to improve MNH services in a sustainable manner in South Africa is still to be evaluated. Our experiences to date demonstrate that inclusivity, embeddedness, responsivity, and agility in the context of crises such as the COVID-19 pandemic are key pillars of interventions aiming to improve MNH services. Data will be published as they emerge on QI team functioning and the measured effectiveness of Mphatlalatsane.

## Supplementary Material

22-00022-Odendaal-Supplement.pdf

## Notes

**Peer Reviewed**

**First published online:** October 21, 2022.
