# Supplementary material for: Early Reflections on Mphatlalatsane, a Maternal and Neonatal Quality Improvement Initiative Implemented During COVID-19 in South Africa
Source: Glob Health Sci Pract. 2022 Oct 31;10(5):e2200022. doi: 10.9745/GHSP-D-22-00022 (PMC9622289; doi:10.9745/GHSP-D-22-00022)
Supplement: 22-00022-Odendaal-Supplement.pdf [file 22-00022-Odendaal-Supplement.pdf]

Cite this article as: Odendaal W, Goga A, Chetty T, et al. Early reflections on Mphatlalatsane, a maternal and neonatal quality improvement initiative implemented during COVID-19 in South Africa. *Glob Health Sci Pract*. 2022;10(5):e2200022. https://doi.org/10.9745/GHSP-D-22-00022

Supplement Table S1. Extract from the Mphatlalatsane COVID-19 risk matrix at management level

Aims	Key Areas	Specific strategies
To provide uninterrupted maternal and newborn health services, with a focus on maternity services	1. Effective leadership and management<br>2. Prevent and manage health care worker infection and emotional distress	• Promote visible, decisive management<br>• Ensure effective communication with health care workers<br>• Provide reliable essential supplies (e.g., personal protective equipment)

Supplement Table S2. Extract from the Mphatlalatsane COVID-19 risk matrix at the health care worker level, using family planning services as an example

Point in the continuum of Maternal and Newborn Health Care	Risks of COVID-19 exposure	Risks to continuity of care and quality		
		**Contraceptive provision**	**Pregnancy and fetus**	**Newborn**
Family planning services	Exposure in queues	Low uptake of family planning services	Adolescent, unwanted, and high-risk pregnancies	Prematurity
